# Nitric Oxide Mediates Root K^+^/Na^+^ Balance in a Mangrove Plant, *Kandelia obovata*, by Enhancing the Expression of AKT1-Type K^+^ Channel and Na^+^/H^+^ Antiporter under High Salinity

**DOI:** 10.1371/journal.pone.0071543

**Published:** 2013-08-19

**Authors:** Juan Chen, Duan-Ye Xiong, Wen-Hua Wang, Wen-Jun Hu, Martin Simon, Qiang Xiao, Juan Chen, Ting-Wu Liu, Xiang Liu, Hai-Lei Zheng

**Affiliations:** 1 Key Laboratory for Subtropical Wetland Ecosystem Research of MOE, College of the Environment and Ecology, Xiamen University, Xiamen, Fujian, China; 2 Laboratory of Biological Resources Protection and Utilization of Hubei Province, Hubei Institutes for Nationalities, Enshi, Hubei, China; 3 Department of Biology, Huaiyin Normal University, Huaian, Jiangsu, P.R. China; Macquarie University, Australia

## Abstract

It is well known that nitric oxide (NO) enhances salt tolerance of glycophytes. However, the effect of NO on modulating ionic balance in halophytes is not very clear. This study focuses on the role of NO in mediating K^+^/Na^+^ balance in a mangrove species, *Kandelia obovata* Sheue, Liu and Yong. We first analyzed the effects of sodium nitroprusside (SNP), an NO donor, on ion content and ion flux in the roots of *K. obovata* under high salinity. The results showed that 100 μM SNP significantly increased K^+^ content and Na^+^ efflux, but decreased Na^+^ content and K^+^ efflux. These effects of NO were reversed by specific NO synthesis inhibitor and scavenger, which confirmed the role of NO in retaining K^+^ and reducing Na^+^ in *K. obovata* roots. Using western-blot analysis, we found that NO increased the protein expression of plasma membrane (PM) H^+^-ATPase and vacuolar Na^+^/H^+^ antiporter, which were crucial proteins for ionic balance. To further clarify the molecular mechanism of NO-modulated K^+^/Na^+^ balance, partial cDNA fragments of inward-rectifying K^+^ channel, PM Na^+^/H^+^ antiporter, PM H^+^-ATPase, vacuolar Na^+^/H^+^ antiporter and vacuolar H^+^-ATPase subunit c were isolated. Results of quantitative real-time PCR showed that NO increased the relative expression levels of these genes, while this increase was blocked by NO synthesis inhibitors and scavenger. Above results indicate that NO greatly contribute to K^+^/Na^+^ balance in high salinity-treated *K. obovata* roots, by activating AKT1-type K^+^ channel and Na^+^/H^+^ antiporter, which are the critical components in K^+^/Na^+^ transport system.

## Introduction

Intracellular K^+^/Na^+^ balance is fundamental to the physiology of living cells and is crucial for plant normal growth [1], [2]. Optimal K^+^/Na^+^ ratio is very important not only for the activities of many cytosolic enzymes, but also for maintaining the ideal osmoticum and membrane potential for cell volume regulation [3]. Nevertheless, high salinity condition disturbs intracellular K^+^/Na^+^ balance and causes ion toxicity and osmotic stress in plants [3].

In order to maintain the optimal cytosolic K^+^/Na^+^ balance and avoid the adverse effects of high salinity on plant growth and development, halophytes have developed different strategies to avoid excessive Na^+^ accumulation and to maintain osmotic balance in plants. A common strategy involves the transport restriction of excess Na^+^ *via* inhibiting non-selective cation channels (NSCCs) in the root cells [4]. Moreover, halophytes can elevate the Na^+^ extrusion from the cytosol to external medium and/or Na^+^ compartmentation into the vacuoles through trans-membrane transport proteins like plasma membrane (PM)-located Na^+^/H^+^ antiporter (SOS1) and tonoplast-located Na^+^/H^+^ antiporter (NHX1) [5], [6]. The process of Na^+^/H^+^ antiporter-mediated Na^+^ extrusion and Na^+^ compartmentation is energy-dependent, and this energy is supplied by the proton-motive force, which can be generated by H^+^-translocating pumps (e.g., H^+^-ATPase and H^+^-PPiase) [7], [8].

As well known, maintaining a optimal K^+^/Na^+^ ratio in the cytoplasm is more important than simply maintaining a low Na^+^ concentration in many plant species under high salinity [2]. Because Na^+^ competes with K^+^ for uptake into roots [3], NaCl-induced K^+^ loss is an important plant response to high salinity [9]. The transcript levels of several K^+^ transport-related genes, such as the shaker K^+^ channel gene and the high affinity K^+^ transport/K^+^ uptake transporter-type gene, are either down- or up-regulated by salt treatment, which probably reflects the different capacities of plants to modulate K^+^ uptake from the roots [3]. Noticeably, the inward-rectifying potassium channels (AKT1), a major route for K^+^ uptake from external environment by root epidermis, exhibited the high K^+^/Na^+^ selectivity at physiological K^+^ and Na^+^ concentrations [10]. Since the first *AKT1* from *Arabidopsis* was cloned in 1992 [11], *AKT1* genes have been identified in many other species, such as *TaAKT1* from *Triticum aestivum* [12], *OsAKT1* from *Oryza sative* [13], *CaAKT1* from *Capsicum annuum* [14] and *PutAKT1* from *Puccinellia tenuiﬂora* [15]. Besides regulating K^+^ uptake, AKT1 is also indirectly involved in SOS pathway [3]. Qi and Spalding [16] found that the defect in Na^+^ efflux in the *sos* mutant could lead to excessive Na^+^ in the cytoplasm that was inhibitory to AKT1, resulting in poor growth due to the impaired K^+^ uptake. Mutant analyses showed that *akt1* mutant was sensitive to salt during early seedling development, indicating that AKT1 played a critical role in maintaining cytoplasmic K^+^/Na^+^ balance in salt-treated plants [17].

Nitric oxide (NO), an important signaling molecule, plays a critical role in wide range of physiological and developmental processes in plants including root formation, seed germination, stomatal closure, pollen tube growth and flowering [18]. Moreover, NO has been demonstrated to be involved in mediating the responses to biotic and abiotic stresses in plants, such as drought, salt, heat stress and disease resistance [19]. It was reported that exogenous NO significantly enhanced salt tolerance in maize seedlings through increasing the activities of H^+^-ATPase and Na^+^/H^+^ antiporter in the tonoplast [20]. NO was also found to serve as a signal for inducing salt resistance by decreasing Na^+^ content in reed callus [21]. Until now, studies were more focused on the effects of NO on avoiding excess Na^+^ accumulation in cytoplast [6], [20], [21]. However, the precise mechanism of K^+^ uptake and cytosolic K^+^/Na^+^ balance modulated by NO is not very clear yet, and the possible pathways for NO signaling that can regulate the expressions and activities of ion transporters to maintain a optimal K^+^/Na^+^ ratio in cytoplasm have not been investigated.

*Kandelia obovata* Sheue, Liu and Yong is the dominant mangrove species in East Asia and is mostly distributed in the regions of estuaries, deltas and riverbanks or along the coastlines of tropical and subtropical areas [22]. In contrast to some mangrove species with special morphological structure, such as salt glands and salt hairs, *K. obovata* is a typical non-secretor mangrove species [22]. *K. obovata* evolved many mechanisms to resist high salinity such as ultrafiltration by roots, osmotic adjustment and regulation of reactive oxygen species [17]. The most striking feature of *K. obovata* is the maintenance of cell turgor by accumulating inorganic ions in the vacuole and organic compounds in the cytoplasm [17]. A previous study found that *K. obovata* could exclude 99% of the salt in surrounding seawater by ultrafiltration [23]. Li et al [24] reported that *K. obovata* exhibited a higher capacity to restrict Na^+^ uptake and accumulation under increasing salt treatments. However, the existing studies were mostly done at physiological and biochemical levels, the molecular mechanisms of salt tolerance in mangrove plants are still not well known. Recently, Lu et al [25] found that NO contributed to maintain ionic homeostasis by increasing Na^+^ efflux and inhibiting K^+^ loss in salt-treated *K. obovata* roots. Our previous study also found that NO could enhance salt secretion from salt glands and promote Na^+^ sequestration into vacuole of another mangrove plant, *Avicennia marina* [6]. Although increasing evidence suggests that NO functions as an important intermediate for regulating ionic homeostasis under salt stress, further investigations are needed to clarify the precise molecular mechanism by which NO regulates K^+^ uptake and K^+^/Na^+^ balance in the roots of mangrove plants.

In the light of the above context, in this study, we first characterized the effects of various concentrations of sodium nitroprusside (SNP, an NO donor) on K^+^ and Na^+^ content, K^+^/Na^+^ ratio, and net K^+^ and Na^+^ fluxes in NaCl-treated *K. obovata* roots using inductively coupled plasma mass spectrometry (ICP-MS), X-ray microanalysis and the scanning ion-selective electrode technique (SIET). Secondly, due to the fact that Na^+^ extrusion from the cytoplast depend on PM H^+^-ATPase as well as Na^+^/H^+^ antiporter, we investigated the protein expressions of H^+^-ATPase and Na^+^/H^+^ antiporter using western blot analysis. Moreover, to further clarify the precise molecular mechanism by which NO regulates K^+^ uptake and K^+^/Na^+^ balance in *K. obovata*, some critical genes related to K^+^/Na^+^ transport system (e.g., *AKT1*, *SOS1*, *NHX1*, etc.) were isolated and the transcriptional expressions of these genes were analyzed using quantitative real-time PCR (qRT-PCR). Based on above experiments, we attempted to clarify the detailed mechanism of NO in modulating cytosolic K^+^/Na^+^ balance, which might be closely related to the activity and expression of K^+^/Na^+^ transport system in salt-treated *K. obovata* roots.

## Results

### Effects of NO on element ratios and distribution in *K. obovata* roots

**Figure 1:**
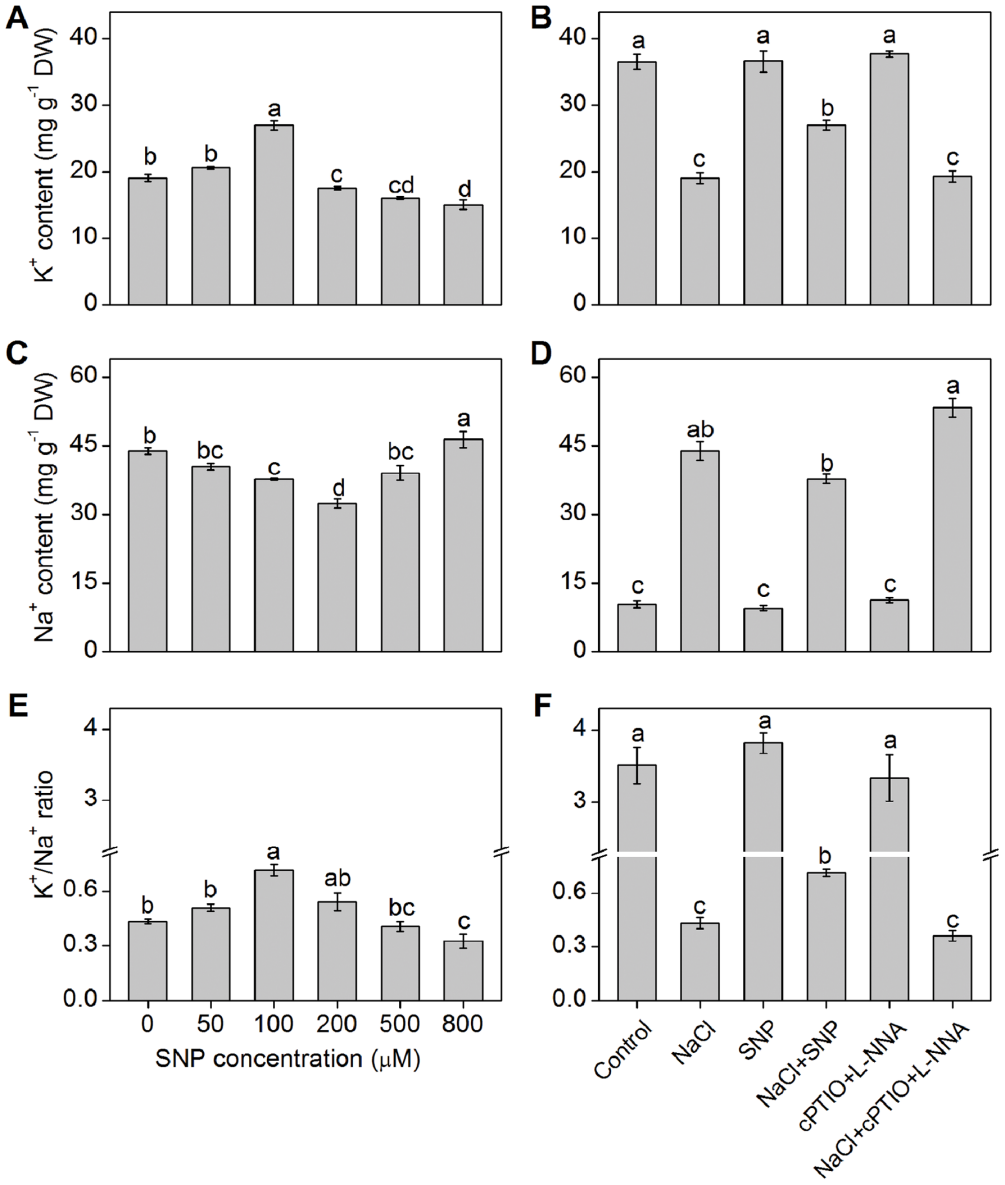
Effects of NO on K^+^, Na^+^ content and K^+^/Na^+^ ratio in *K. obovata* roots. K^+^ content (A), Na^+^ content (C) and K^+^/Na^+^ ratio (E) in *K. obovata* roots after 15 days of 400 mM NaCl and different concentrations of SNP treatment. Shown in (B), (D) and (F), respectively, show K^+^ content, Na^+^ content and K^+^/Na^+^ ratio in roots of *K. obovata* seedlings treated for 15 days with the following Hoagland solution-based treatments: Control (Hoagland solution), NaCl (400 mM), SNP (100 µM), NaCl + SNP (400 mM NaCl+100 µM SNP), cPTIO+L-NNA (200 µM cPTIO+100 µM L-NNA), NaCl+cPTIO+L-NNA (400 mM NaCl+200 µM cPTIO+100 µM L-NNA). Each column is the mean value of at least three independent seedlings and bars represent the SE of the mean. For each parameter, bars with different letters indicate significantly different at the 0.05 level.

After *K. obovata* seedlings were treated with 400 mM NaCl and various concentrations of SNP for 15 days, the appropriate concentration of exogenous NO (100 µM SNP) significantly increased K^+^ content and decreased Na^+^ content, resulting in the increased K^+^/Na^+^ ratio compared with the solely NaCl-treated seedlings (Figures 1A, C and E). According to this result, 100 µM SNP, which could provide an optimal NO concentration for maintaining ionic balance in *K. obovata* seedlings, was used in the following experiments. To attribute the role of NO in enhancing K^+^/Na^+^ ratio in *K. obovata* roots exposed to high salinity, specific NO synthesis inhibitor (L-NNA) and NO scavenger (cPTIO) were used in the next experiments. As shown in Figures 1B, D and F, both L-NNA and cPTIO abolished the effects of NO and caused a decrease in K^+^ content and K^+^/Na^+^ ratio, but an increase in Na^+^ content in salt-treated *K. obovata* roots. Moreover, the results of X-ray microanalysis showed that 100 µM SNP significantly increased K^+^ percentage, meanwhile decreased Na^+^ percentage, resulting in the higher K^+^/Na^+^ ratio in both cortex and stele cells of *K. obovata* roots (Table 1). Remarkably, K^+^/Na^+^ ratio in stele cells was markedly higher than that in the cortex under various SNP concentrations (Table 1).

**Table 1:** Table 1. Effects of NO on the percentage of K^+^, Na^+^ and K^+^/Na^+^ ratio in cortex and stele section of *K. obovata* roots under 400 mM NaCl treatment for 15 days.

Measuring region		SNP concentration (μM)		
		0	100	800
	K^+^	20.2±2.3^a^	21.9±1.2^a^	15.7±3.1^b^
Cortex	Na^+^	30.5±1.7^ab^	25.0±4.2^b^	42.3±2.6^a^
	K^+^/Na^+^	0.7±0.1^b^	0.9±0.1^c^	0.4±0.1^a^
	K^+^	23.1±2.2^c^	40.1±4.5^a^	31.3±4.7^b^
Stele	Na^+^	32.3±1.6^b^	22.8±1.7^c^	42.0±3.5^a^
	K^+^/Na^+^	0.7±0.1^a^	1.8±0.1^c^	0.8±0.1^b^

The ratio of elements in roots was measured using the X-ray microanalysis. The results were calculated by expressing the percentage of atomic number for a particular element (K^+^ or Na^+^) in the total atomic number for all the elements (K^+^, Na^+^, Ca^2+^, Mg^2+^, Al^3+^ and Mn^2+^) detected in the given region of the root samples. Each data are the means of 5–8 measurements. Values followed by the same letter in a line are not significantly different (*P*>0.05) as described by one-way ANOVA.

### Effects of NO, specific inhibitor and NO scavenger on K^+^ and Na^+^ fluxes in *K. obovata* roots

**Figure 2:**
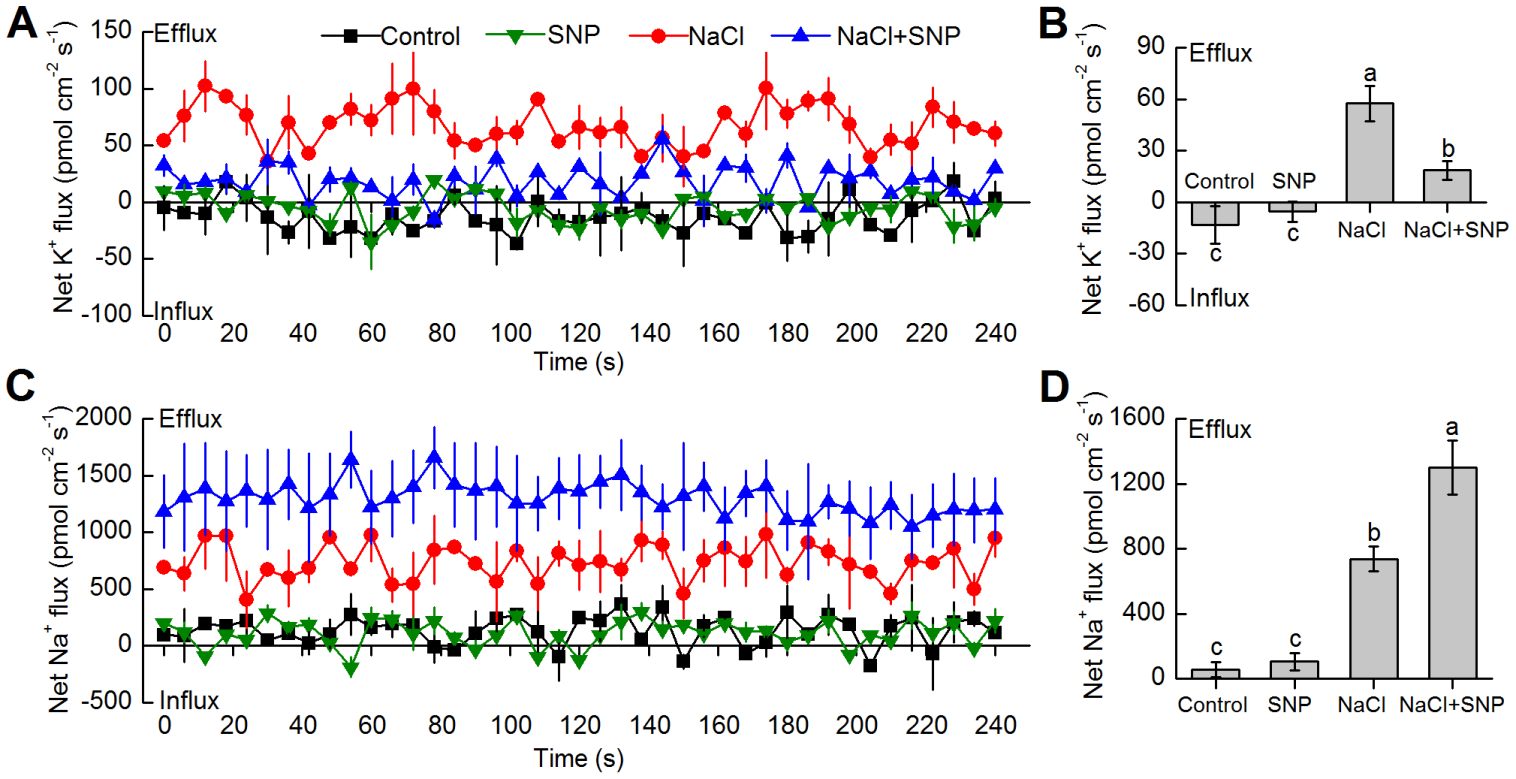
Effects of NaCl and SNP on K^+^ and Na^+^ fluxes in *K. obovata* roots. After 15 days of exposure to NaCl (400 mM), SNP (100 µM) and NaCl (400 mM)+SNP (100 µM), the net K^+^ (A) and Na^+^ (C) fluxes from the mature root zone (20 mm from the tip) of *K. obovata* seedlings. The positive value in the figures represents the net influx and negative value represents the net efflux. Each point represents the mean value of four individual roots and bars represent the SE of the mean. The mean fluxes of K^+^ (B) and Na^+^ (D) within the measuring periods are shown. Columns labeled with different letters indicates significantly different at *P*<0.05.

As shown in Figures 2A and C, the control plants grown in Hoagland solution alone exhibited the slightly inward K^+^ flux and outward Na^+^ flux in *K. obovata* roots, although the flux rates oscillated during the period of recording. Compared with the control plants, 100 μM SNP did not significantly change K^+^ and Na^+^ flux profiles in *K. obovata* roots under no-salt condition (Figure 2). However, after *K. obovata* seedlings were treated with 400 mM NaCl for 15 days, the rectifications of K^+^ flux in the root tips exhibited the evident efflux and reached the maximal mean K^+^ efflux of 58 pmol cm^–2^s^–1^ (Figure 2B). It is noteworthy that the K^+^ efflux in *K. obovata* roots under 100 μM SNP and 400 mM NaCl treatment for 15 days were inhibited, which was about 72% lower than that under solely NaCl-treated plants (Figures 2A and B).In contrast to the results of net K^+^ flux, net Na^+^ efflux in NaCl-treated *K. obovata* roots significantly enhanced and reached a mean rate of 737 pmol cm^–2^s^–1^, which was about 5-fold higher than that of the control (Figures 2C and D). After the plants were treated with 100 µM SNP and 400 mM NaCl for 15 days, net Na^+^ efflux in *K. obovata* roots increased by a maximal mean rate of 1300 pmol cm^–2^s^–1^ (about 10-fold higher than that with control condition) (Figures 2C and D).

**Figure 3:**
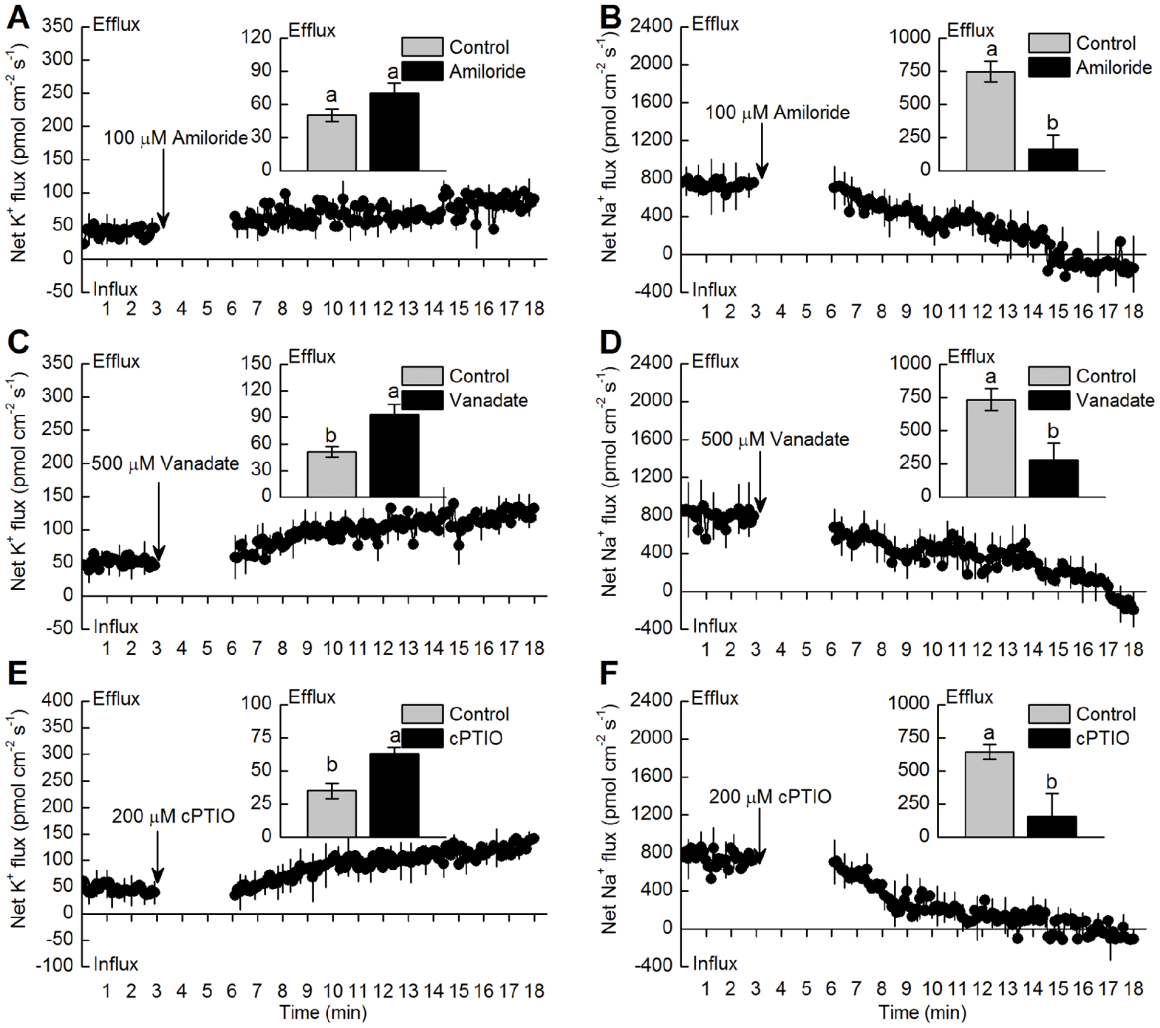
Changes in net K^+^ and Na^+^ fluxes after specific inhibitors and NO scavenger addition. Effects of specific Na^+^/H^+^ antiporter inhibitor (100 µM amiloride, A and B), H^+^-ATPase inhibitor (500 µM vanadate, C and D) and NO scavenger (200 µM cPTIO, E and F) on the net K^+^ and Na^+^ fluxes in the mature root zone (20 mm from the tip) of *K. obovata* seedlings with 400 mM NaCl treatment for 15 days. The positive value in the figures represents the net influx and negative value represents the net efflux. The mean K^+^ or Na^+^ flux within the measuring periods is shown in the inserted section. Each point represents the mean value of four individual roots and bars indicate the SE of the mean. Before the addition of specific inhibitor or NO scavenger, steady K^+^ or Na^+^ flux was measured for about 3 min. Column labeled with different letters are significantly different at *P*<0.05.

Due to the fact that intracellular ionic balance is closely related to the activity of H^+^-ATPase and Na^+^/H^+^ antiporter, H^+^-ATPase inhibitor (vanadate) and Na^+^/H^+^ antiporter inhibitor (amiloride) were used for clarifying the critical role of H^+^-ATPase and Na^+^/H^+^ antiporter in regulating K^+^ and Na^+^ fluxes. Figures 3A and C show that compared with the mean value of Na^+^ flux before inhibitor addition, transient application of both 100 µM amiloride and 500 µM vanadate increased net K^+^ efflux in the root tips of *K. obovata* seedlings under 400 mM NaCl treatment for 15 days. Unlike K^+^ efflux, the addition of specific inhibitors decreased net Na^+^ efflux (Figures 3B and D). Additionally, cPTIO, a specific NO scavenger, was applied to test the effects of NO on K^+^ and Na^+^ fluxes in NaCl-treated *K. obovata* roots. Similar to the patterns of ion flux affected by amiloride and vanadate, cPTIO also accelerated K^+^ efflux and inhibited Na^+^ efflux, as compared to the mean value of ion flux before cPTIO addition (Figures 3E and F).

### Effects of NO on the protein expression of H^+^-ATPase and Na^+^/H^+^ antiporter

**Figure 4:**
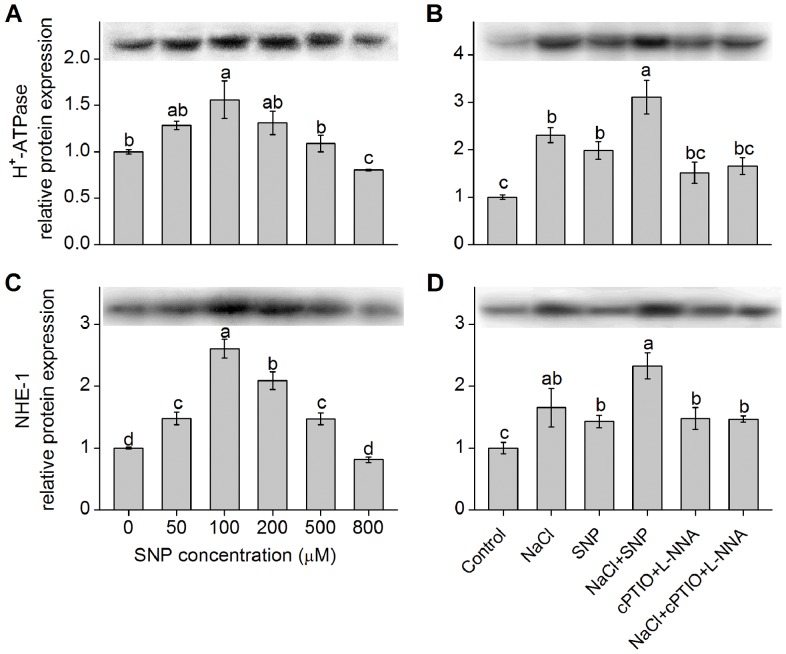
Western blot analysis of protein expression of plasma membrane (PM) H^+^-ATPase and Na^+^/H^+^ antiporter (NHE-1). Shown in (A) and (C) are the relative protein expression level of *K. obovata* seedlings grown under 400 mM NaCl and various concentrations of SNP for 15 days. Shown in (B) and (D) represent the relative protein expression level of *K. obovata* seedlings treated with the following Hoagland solution-based solutions for 15 days: Control (only Hoagland solution), NaCl (400 mM), SNP (100 µM), NaCl+SNP (400 mM NaCl+100 µM SNP), cPTIO+L-NNA (200 µM cPTIO+100 µM L-NNA), NaCl+cPTIO+L-NNA (400 mM NaCl+200 µM cPTIO+100 µM L-NNA). Three independent experiments were performed. The relative intensity of bands (column charts) was quantified by densitometric analysis with Quantity One software and expressed relative to the intensity of the control band defined as 1. For each parameter, bars with different letters indicate significant difference at *P*<0.05.

Western-blot analysis exhibited that the protein expression level of H^+^-ATPase and Na^+^/H^+^ antiporter reached the maximum value in NaCl-treated *K. obovata* roots in the presence of 100 µM SNP (Figures 4A and C). However, compared with NaCl treatment, the increased protein expression of H^+^-ATPase and Na^+^/H^+^ antiporter induced by SNP was completely reversed by cPTIO and L-NNA addition (Figures 4B and D).

### Effects of NO on transcriptional expression of several K^+^/Na^+^ balance related genes

**Figure 5:**
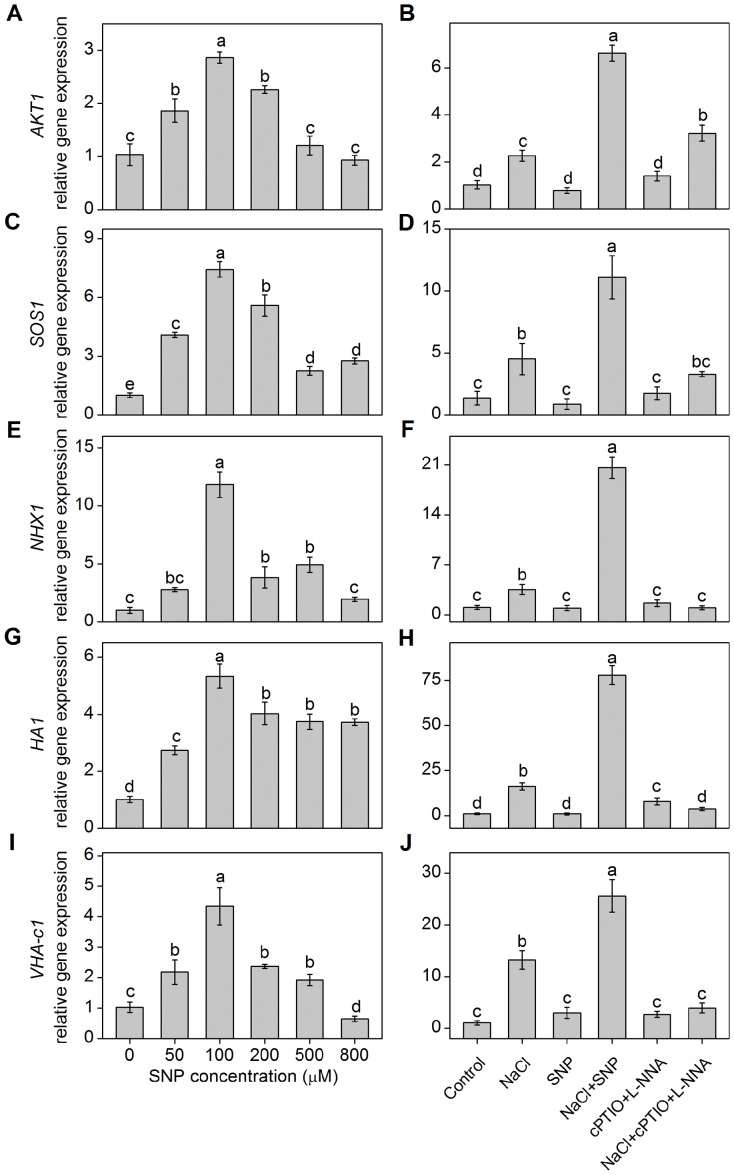
Effects of NO on transcriptional expression of several K^+^/Na^+^ balance related genes. Expressions of *AKT1* (A), *SOS1* (C), *NHX1* (E), *HA1* (G) and *VHA-c1* (I) genes in *K. obovata* roots in response to 400 mM NaCl and various concentrations of SNP for 15 days. Shown in B, D, F, H and J, respectively, stand for the gene expressions of *AKT1*, *SOS1*, *NHX1*, *HA1*, *VHA-c1* in the roots of *K. obovata* seedlings grown under the following Hoagland solution-based solutions for 15 days: Control (only Hoagland solution), NaCl (400 mM), SNP (100 µM), NaCl + SNP (400 mM NaCl+100 µM SNP), cPTIO+L-NNA (200 µM cPTIO+100 µM L-NNA), NaCl+cPTIO+L-NNA (400 mM NaCl+200 µM cPTIO+100 µM L-NNA). The relative transcriptional expression of each gene was performed by normalization with *18S* rRNA, a reference gene of *K. obovata*. Mean values and SE were calculated from three independent experiments. For each parameter, bars with different letters indicate significant difference at *P*<0.05.

The qRT-PCR analysis showed that 100 µM SNP remarkably up-regulated the relative expression levels of *AKT1* gene, which was about 4-fold higher than those of the control (0 µM SNP) (Figure 5A). Similarly, the relative expression levels of *SOS1*, *NHX1*, *HA1* and *VHA-c1* gene in 100 µM SNP-treated plants were also significantly higher than those in other treatments. In agreement with this, NO synthesis inhibitor (L-NNA) and NO scavenger (cPTIO) down-regulated the expression levels of *AKT1*, *SOS1*, *NHX1*, *HA1* and *VHA-c1* gene and reversed the effects of NO (Figure 5).

## Discussion

### NO modulates K^+^/Na^+^ balance by decreasing K^+^ loss and increasing the expression of AKT1

There is no doubt that K^+^ plays a vital role in salt adaptation of plants [3]. Under normal physiological conditions, the cytosolic K^+^ is maintained at a steady state level [26]. However, under high salinity condition, due to physicochemical similarities between K^+^ and Na^+^, excess Na^+^ tends to substitute K^+^ and hence impairs cellular biochemistry [27]. Previous study reported that a strong correlation existed between the ability of plants to retain K^+^ and salt tolerance [9]. Carden et al [28] also found that the barley variety with strong salt tolerance could better maintain root cytosolic K^+^ under high-NaCl treatment. It should be noted that maintaining the steady K^+^ level is also critical in halophytes [29]. For instance, Hariadi et al [29] found that shoot sap K^+^ content increased along with the increased salt content in *Chenopodium quinoa* leaves. Lu et al [25] reported that salinity caused an evident K^+^ efflux in the roots of two mangrove species, *Bruguiera gymnorrhiza* and *K. obovata*, which was similar to our study showing that K^+^ efflux remarkably increased in NaCl-treated *K. obovata* roots (Figures 2C and D), indicating that K^+^ plays an important role in osmotic adjustment under high salinity. Thus, minimizing NaCl-induced K^+^ loss appears to be critical for enhancing salt tolerance of plants. NO, as a signaling molecule, is involved in the resistant response of plants to high salinity [19]. Zhao et al [21] found that NO enhanced salt resistance by increasing K^+^/Na^+^ ratio in the reed callus. Consistent with the previous study, application of SNP at medium concentrations (100 µM) significantly increased K^+^ content and K^+^/Na^+^ ratio (Figures 1A and E; Table 1), while NO synthesis inhibitor and NO scavenger reversed the effects of NO, indicating that NO plays an important role in maintaining K^+^ homeostasis in high salinity-treated *K. obovata* roots.

It is widely established that under high salinity condition, the PM is strongly depolarized, which causes the remarkable K^+^ loss *via* depolarization-activated (DA) channels, e.g., NSCCs and KORCs (outward rectifying K^+^ channels) in some plants (e.g., barley, *Arabidopsis*, etc.) [1]. The higher H^+^-ATPase activity can lead to hyperpolarization or repolarization of the PM, thus limit the K^+^ efflux through DA-NSCC and DA-KORC [30]. In this study, salt shock-induced K^+^ efflux in *K. obovata* roots was markedly enhanced by vanadate, an inhibitor of PM H^+^-ATPase (Figure 3C). This finding is similar to recent reports indicating that the capacity to maintain low K^+^ efflux and retain K^+^ is highly dependent on an active PM H^+^-ATPase in *Populus euphratica* and *K. obovata* [8], [25]. It should be noted that NO could increase K^+^ percentage and K^+^/Na^+^ ratio in NaCl-treated *Arabidopsis*, which was dependent on the increased PM H^+^-ATPase activity [21]. For *K. obovata* under salt condition, previous study found that the decreased K^+^ efflux induced by NO likely accounted for the less depolarized membrane potential maintained by high activity of PM H^+^-ATPase [25]. Similarly, NaCl-induced transient K^+^ efflux in the root tips of *K. obovata* was remarkably inhibited by PM H^+^-ATPase inhibitor (Figure 3C), the protein and gene expressions of PM H^+^-ATPase were significantly up-regulated *K. obovata* roots due to NO application in this study (Figures 4 and 5). These results suggested that the increased H^+^-ATPase activity, which was induced by NO, might be necessary for decreasing the salt-induced K^+^ efflux and for restricting K^+^ loss in *K. obovata* grown under high salinity [1], [8].

AKT1, the most important inward-rectifying shaker K^+^ channel, whose gene is mainly expressed in root epidermal cells, is involved in K^+^ acquisition by plants [15]. It’s known that this type of channel plays an important role in K^+^ uptake from the soil solution and greatly attributes to maintain and restore K^+^ homeostasis in high salinity-treated plants [31]. The expression of *AKT1* gene showed different responses to salinity in plants with different salt tolerance. Fuchs et al [32] reported that salt stress strongly down-regulated the transcriptional expression of *OsAKT1* gene in *Oryza sativa*, and a similar response was also found in *Arabidopsis* [33]. In contrast, the expression levels of the AKT1-type K^+^ channel gene from *Puccinellia tenuiflora*, *PutAKT1*, seem to be unaffected by excessive external NaCl, and overexpression of *PutAKT1* significantly enhanced the resistence to high salinity in transgenic *Arabidopsis* [15]. Interestingly, previous study found that the *Arabidopsis sos* mutant had a growth defect under K^+^-limiting conditions [3]. They speculated that the decreased Na^+^ efflux in the *sos* mutant may result in high Na^+^ accumulation in cytoplast, which may indirectly inhibit the activity of K^+^ transporter such as AKT1 and thus regulate K^+^ uptake in the roots [3]. In the present work, the transcriptional expression of *AKT1* was up-regulated in roots of NaCl-treated *K. obovata* (Figure 5B). The likely reason is that glycophytes (e.g., *Arabidopsis* and *O. sativa*) mainly grows in the soils with low salinity, so their mechanisms developed for K^+^ uptake and transport may not operate as efficiently as in halophytes under high salinity condition. Most importantly, we found that SNP significantly up-regulated the transcriptional expression of *AKT1* gene in the roots of NaCl-treated *K. obovata* (Figures 5A and B), indicating that NO might play an important role in increasing K^+^ uptake by inducing the expression of *AKT1* gene in salt-treated *K. obovata* roots. AKT also plays an important role in K^+^ circulation through the xylem and phloem [34]. In this study, SNP significantly increased K^+^ percentage in the stele cells (Table 1), indicating that NO-modulated K^+^ accumulation and distribution in the roots may be related to the activity and expression of AKT1-type K^+^ channel.

### NO modulates K^+^/Na^+^ balance by increasing Na^+^ extrusion

Maintaining a low Na^+^ content in the cytoplasm is a key factor for cellular adaptation to high salinity condition [35]. Zhao et al [21] reported that NO served as a signal that induced salt resistance by decreasing Na^+^ content and increasing K^+^/Na^+^ ratio in reed callus, which is dependent on the increased PM H^+^-ATPase activity. In this study, we also confirmed that NO could decrease intracellular Na^+^ accumulations and K^+^/Na^+^ ratio, whereas the treatment with NO synthesis inhibitor (L-NNA) and specific scavenger (cPTIO) completely blocked the effects of NO on element ratio (Figure 1 and Table 1), which were in accordance with previous results from Zhao et al [21]. SIET data showed that 100 µM SNP significantly enhanced net Na^+^ efflux in salt-treated *K. obovata* roots (Figure 2A), while the inhibitors of PM Na^+^/H^+^ antiporter, H^+^-ATPase and NO scavenger significantly decreased net Na^+^ efflux (Figure 3A, C and E). These findings are similar to previous results showing that Na^+^ efflux were enhanced by NO in *B. gymnorrhiza* and *K. obovata* through regulation of Na^+^/H^+^ antiporter and H^+^-ATPase in the PM [25]. It is clear that NO functions in maintaining lower Na^+^ content in *K. obovata* roots by increasing Na^+^ extrusion to the external environment. However, NO is thought to be a “double-edged sword”, because it is a reactive nitrogen species and readily reacts with the superoxide anion-radical (O_2_^–^). The formation of peroxynitrite ion (ONOO^–^) could cause serious damage to cell structures [36]. Therefore, the protection or toxicity effect of NO depends on its concentration and the plants developmental stages [6], [20]. For instance, high SNP concentration (800 μM) induced the obvious increase in K^+^/Na^+^ ratio (Figure 1E), which may exaggerate the high salinity-induced damage in *K. obovata*.

At the cellular level, in order to maintain ion homeostasis, PM Na^+^/H^+^ antiporter, which is driven by PM H^+^-ATPase, is important for removing excess Na^+^ from the cytoplasm to the apoplast [3], [37]. Recent research reported that up-regulation of PM H^+^-ATPase activity facilitated Na^+^ efﬂux into the apoplast [20], [21], contributing to alleviation of Na^+^ toxicity in plants under salt stress [38]. The PM Na^+^/H^+^ antiporter encoded by *SOS1* has been suggested to be related to the long distance Na^+^ transport and to Na^+^ extrusion from meristem zone of the roots to external medium under high salinity [3], [37], [39]. Shi et al [37] confirmed that the *sos1* mutants are extremely sensitive to high concentration of external Na^+^, and over-expression of Na^+^/H^+^ antiporter gene (*AtSOS1*) markedly improved salt tolerance in *Arabidopsis*. Importantly, previous studies have reported that NO alleviated salt toxicity by increasing the activities of H^+^-ATPase [20], [21]. Our previous results also suggested that the salt secretion in the salt glands of *A. mari*na, which was enhanced by NO, was closely related to the increased expression levels of *HA1* and *SOS1* genes [6]. Similarly, in the present study, 100 µM SNP remarkably increased the expression of Na^+^/H^+^ antiporter and H^+^-ATPase at both protein and gene levels in high salinity-treated *K. obovata* roots (Figures 4 and 5), which may greatly contribute to maintain lower Na^+^ level in the cytoplast.

Moreover, Na^+^ compartmentation is another efficient strategy for plant cells to cope with high salinity conditions [40]. The tonoplast Na^+^/H^+^ antiporter (NHX) and H^+^-ATPase (VHA-c) function in mediating Na^+^ sequestration into the vacuoles [41]. In accordance with the results of our previous study on *A. marina* [6], this study found that SNP significantly up-regulated the transcriptional level of *NHX1* and *VHA-c1* genes in salt-treated *K. obovata* roots (Figures 4 and 5). This result implies that NO might contribute to modulation of Na^+^ compartmentation into the vacuoles, the detailed mechanism for this role of NO deserves further attention.

### Pathway of NO-regulated K^+^/Na^+^ balance in salt-treated *K. obovata* root

**Figure 6:**
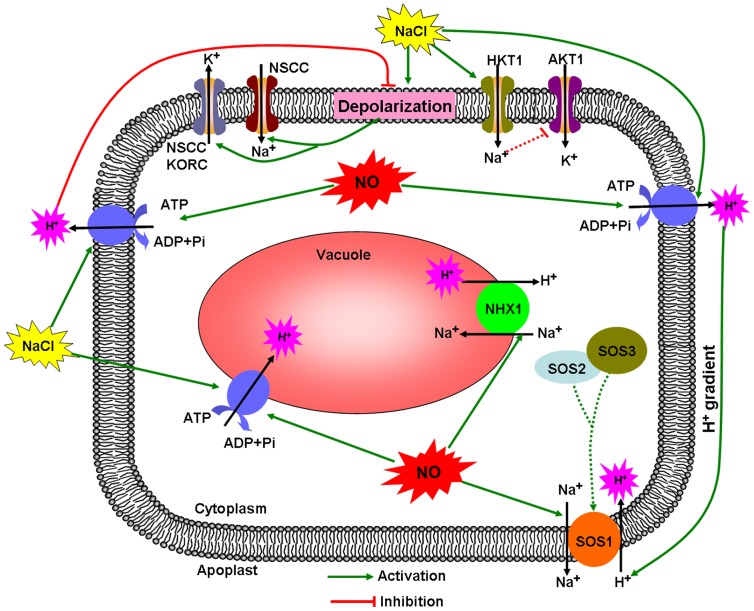
A schematic model for NO-modulated cellular K^+^/Na^+^ balance in *K. obovata* under high salinity condition. The arrows in solid lines denote the identified regulation. The hypothetical regulation is indicated by broken lines ending in t-bars.

Based on our results and current knowledge about the mechanisms of salt tolerance in plants, we propose a signaling pathway by which NO mediates K^+^/Na^+^ balance in salt-treated *K. obovata.* As shown in Figure 6, high salinity strongly depolarizes the PM and causes excessive Na^+^ influx to the cytoplasm *via* NSCCs, simultaneously enhances K^+^ efflux through depolarization-activated channels such as KORCs and NSCCs. As a result, the excessive Na^+^ restricts K^+^ absorption by competing with the site of K^+^ uptake. Compared with no-salt condition, both high salinity and NO can enhance H^+^ extrusion by activating PM H^+^-ATPase. The roles of up-regulated PM H^+^-ATPase in regulating ionic homeostasis are manifold (Figure 6). Firstly, the increased H^+^ extrusion by H^+^-ATPase contributes to inhibit salt-induced PM depolarization, thus restricting K^+^ efflux by KORCs and NSCCs [4]. Secondly, the enhanced H^+^ pumping can sustain H^+^ gradient, which is necessary for Na^+^ extrusion out of the cytosol by Na^+^/H^+^ antiporter (SOS1) across the PM [42]. In addition, NO can also directly activate PM Na^+^/H^+^ antiport system and contribute to Na^+^ efflux. Interestingly, the intracellular Na^+^ participated in the potential negative regulation of AKT1 activity, implying that the lower cytoplasmic Na^+^ level induced by NO may indirectly enhance K^+^ uptake by some PM K^+^ transporters such as AKT1, which can further increase the cytoplasmic K^+^ level and maintain the K^+^/Na^+^ balance in salt-treated *K. obovata*.

In conclusion, this study provides the evidence that NO functions in decreasing the cytosolic K^+^ loss and increasing the ability of K^+^ uptake in salt-treated *K. obovata* through increasing the expression of PM H^+^-ATPase and AKT1. Moreover, NO can also increase the protein and gene expression of H^+^-ATPase and Na^+^/H^+^ antiporter, which further enhance Na^+^ extrusion in salt-treated *K. obovata* roots. It is clear that NO modulates K^+^/Na^+^ balance in cytoplasm and further enhances salt tolerance of *K. obovata* through increasing the expression of some critical components in K^+^/Na^+^ transport system including AKT1-type K^+^ channel, H^+^-ATPase and Na^+^/H^+^ antiporter.

## Materials and Methods

### Plant materials and culture conditions

In May 2011, the mature propagules of *K. obovata* were collected from Mangrove Nature Reserve at Jiulongjiang River (latitude 24°24′N and longitude 117°23′E) in Fujian Province, China. Four healthy propagules of similar size were randomly planted in individual pots (15 cm in diameter and 18 cm in height) containing clean sand and were cultured in a greenhouse. The photosynthetically active radiation (PAR) was 800–1000 μmol m^–2^ s^–1^, relative humidity was maintained at 60–70%, and daily temperature was 25–28°C. Seedlings were irrigated with tap water every 3 days and the pots were fertilized with a full-strength Hoagland solution biweekly as described by Chen et al [6]. NaCl and SNP treatments were started when the 4th pair of leaves came out from the apex of the growing shoot.

### Treatments

Seedlings of uniform size were chosen and divided into two groups. As described by Kao et al [43] and Li et al [24], 400 mM NaCl, which significantly elevated Na^+^ contents in the root and shoot tissues and inhibited the photosynthesis and growth of *K. obovata* seedlings, was considered as a high salinity for *K. obovata.* Therefore, 400 mM NaCl was used for salinity treatment in this study. In order to select a suitable concentration of SNP to use in the following experiments, different amounts of SNP (0, 50, 100, 200, 500, 800 µM) were added to the Hoagland solution containing 400 mM NaCl in the first group. NO synthesis inhibitor (*N*-nitro-L-arginine, L-NNA) and NO scavenger (2-(4-carboxyphenyl)-4,4,5,5- tetramethylimidazoline-1-oxyl-3-oxide, cPTIO) were used in the second group to clarify the role of NO. The second group consisted of a control (only Hoagland solution without NaCl and SNP) and five Hoagland solution-based treatments as follows: (1) NaCl, 400 mM NaCl; (2) SNP, 100 µM SNP; (3) NaCl+SNP, 400 mM NaCl+100 µM SNP; (4) cPTIO+L-NNA, 200 µM cPTIO+100 µM L-NNA; (5) NaCl+cPTIO+L-NNA, 400 mM NaCl+200 µM cPTIO+100 µM L-NNA. The treatment solutions were renewed three times a week to maintain the constant concentrations. After various treatments for 15 days, some fresh roots of *K. obovata* were collected and immediately used for K^+^ and Na^+^ content analysis, X-ray microanalysis and net K^+^ and Na^+^ efflux measurements. Other plant materials were immediately frozen in liquid nitrogen and stored at –80°C for further analyses such as western-blot and qRT-PCR.

### Measurement of Na^+^ and K^+^ contents

The roots of *K. obovata* were collected and oven-dried for 48 h at 75°C. The dried samples (200 mg) were ashed for 12 h at 450°C in the muffle furnace and then dissolved in 1 ml HNO_3_. After digestion, the acid solution was diluted to an appropriate volume with the deionized water. K^+^ and Na^+^ concentrations in the solution were determined by inductively coupled plasma mass spectrometry (ICP-MS, Perkin Elmer, Inc., Elan DRC-e).

### X-ray microanalysis

Fresh roots of *K. obovata* were cut into 2–4 mm segments from tip with a razor blade and fixed immediately with 2.5% (w/v) glutaraldehyde for 5 h at 4°C. The samples were then rinsed with 0.1 M phosphate buffer solution (pH 7.0) before being dehydrated in a series of alcohol concentrations (50, 70, 80, 90, 95 and 100%) for 10 min. Then, standard procedures like freeze-drying, infiltration and polymerization as described by Fritz [44] were followed. Relative amount of elements in cortical and stellar cell of root tips were analyzed by scanning electron microscope (JSM6390, JEOL, Kyoto, Japan) equipped with an energy dispersive X-ray detector (Kenex, Valencia, CA, USA) [45]. Relative amount of K^+^ and Na^+^ levels was expressed as a percentage of the total atomic number for all the elements (K^+^, Na^+^, Ca^2+^, Mn^2+^, Mg^2+^ and Al^3+^) that were detected from the root sections.

### Measurement of net K^+^ and Na^+^ fluxes

Non-invasive Micro-test Technique (NMT) is a new method to measure non-invasively fluxes of specific ions and/or molecules inward or outward from cell without touching the sample by a micro-electrode. As one of the NMT, Scanning Ion-selective Electrode Technique (SIET) has been widely used in plant research [46]. In this study, net K^+^ and Na^+^ fluxes in the root tips of *K. obovata* were measured using SIET system (BIO-001A, Younger USA Sci. and Tech. Corp., Amherst, MA, USA) as described previously by Sun et al [8] and Chen et al [6]. Details concerning the principles of this method and preparation of ion-selective electrodes were available in previous publications [6], [8]. In order to avoid the mechanical disturbance by the electrode movement, the K^+^ and Na^+^ fluxes must be measured slowly at approximately 1–2 s per point [8]. Ion-selective electrodes were calibrated before and after flux measurements in the following solutions containing different concentration of K^+^ and Na^+^: (1) 0.05, 0.1 and 0.5 mM K^+^; (2) 0.5 and 5 mM Na^+^. The final K^+^ and Na^+^ fluxes were calculated by Fick’s law of diffusion as described by Xu et al [47]. According to Sun et al [8], the net ion influx is represented with positive value and net ion efflux with negative value. At least four individual roots were measured in an independent experiment. Each point in Figure 3 represents the mean ion flux of four individual roots. Data and image acquisition, preliminary processing, control of the electrode positioner and stepper-motor-controlled fine focus of the microscope stage were performed using Mage Flux software (http://www.youngerusa.com/mageﬂux or http://xuyue.net/mageﬂux) [4], [8].

### Steady-state SIET measurement of ion flux

The root segments with ca. 10 mm apices were sampled from the control, NaCl− and NaCl+SNP-treated plants at day 15. To avoid the effects of preloaded ion diffused from the surface of high salinity-treated *K. obovata* roots on ion flux measurement, according to the methods of Sun et al [8], the roots were rinsed with double-distilled water and immediately incubated in the measuring solution (0.1 mM KCl, 0.1 mM CaCl_2_, 0.1 mM MgCl_2_, 0.5 mM NaCl, 0.2 mM Na_2_SO_4_, 0.3 mM MES, pH 6.0) to equilibrate for 30 min. Afterwards, equilibrated samples were immobilized at the bottom of the measuring chamber, a 3 cm diameter plastic dish containing 2–3 ml of fresh measuring solution [6]. Net K^+^ and Na^+^ fluxes were measured in steady conditions for 10 to 15 min to insure that no fluctuation was present.

### Transient K^+^ and Na^+^ Flux kinetics

The root tips of *K. obovata* seedlings grown in 400 mM NaCl were sampled and equilibrated in measuring solution for 30 min. After steady K^+^ and Na^+^ fluxes were recorded for 5–6 min, the kinetics K^+^ and Na^+^ fluxes affected by the inhibitors of PM H^+^-ATPase and Na^+^/H^+^ antiporter and specific NO scavenger were examined by transiently adding sodium orthovanadate (100 μM), amiloride (500 μM) and cPTIO (200 μM), respectively. Steady fluxes of K^+^ and Na^+^ were recorded at least 5 min before addition of the inhibitor or scavenger. Then net K^+^ and Na^+^ fluxes were measured for another 20 min after the inhibitor or scavenger was added to the measuring solution. The data for the first 2–3 min were discarded due to diffusion effects after addition of SNP or inhibitor. All of the K^+^ and Na^+^ fluxes measurements were accomplished at Xuyue Science and Technology Company (Beijing, China).

### SDS-PAGE and western-blot analysis

The root tips (1 g) of *K. obovata* were used for proteins extraction according to the method of Chen et al [6]. As described by Bradford [48], the protein concentrations were quantified using bovine serum albumin as a standard. For western-blot analysis, total proteins (150 µg from each sample) were separated by sodium dodecyl sulfate-polyacrylamide gel electrophoresis (SDS-PAGE) using 12% (w/v) acrylamide gels. After electrophoresis, the separated proteins were transferred to polyvinylidene difluoride (PVDF) membrane for 50 min. The membrane was blocked overnight at 37°C in Western Blocking Buffer (TIANGEN, China). The protein blot was probed with primary antibody of H^+^-ATPase (AS07260, Agrisera, Sweden) or Na^+^/H^+^ antiporter (AS09484, Agrisera, Sweden), each diluted at the proportion of 1:2000 and agitated at room temperature for 3 h. Blots were then washed three times in phosphate buffered saline with Tween-20 solution (PBST) containing 50 mM Tris-HCl (pH 8.0), 150 mM NaCl, 0.05% Tween-20 (v/v), and followed by incubation with the secondary antibody (anti-rabbit IgG horse radish peroxidase-conjugated; Abcam, UK, 1∶5000 dilution) for 2 h at room temperature. Finally, the blots were developed with ECL substrate detection solution (TIANGEN, China). Images of the blots were obtained using a CCD imager (FluorSMax Bio-Rad, Hercules, CA, USA). The Quantity One software (Bio-Rad, Hercules, CA, USA) was used to quantify the optical density value of the protein signals.

### Total RNA extraction and gene cloning

Total RNA was isolated from the root tips of *K. obovata* with RNA purification reagents (Invitrogen Inc., CA, USA) according to the manufacturer’s instructions. The RNA integrity and quality were confirmed by 1% agarose gel electrophoresis and ultraviolet spectrophotometer (Cary 50, Varian, USA), respectively. Total RNA (5 μg) was used for the first-strand cDNA synthesis using M-MLV reverse transcriptase (TaKaRa, Dalian, China). For the genes of inward-rectifying potassium channel (*AKT1*), PM Na^+^/H^+^ antiporter (*SOS1*), vacuolar Na^+^/H^+^ antiporer (*NHX1*), PM H^+^-ATPase (*HA1*) and vacuolar H^+^-ATPase subunit c (*VHA* ***-*** *c1*), the reverse transcription products were partially amplified by reverse transcription PCR using the degenerate primers and optimize reaction conditions as shown in Table S1. According to the methods of Chen et al [6], after analyzing the sequence of amplified fragments, partial sequences of *AKT1*, *SOS1*, *NHX1*, *HA1* and *VHA* ***-*** *c1* gene were acquired according to NCBI information (http://www.ncbi.nlm.nih.gov/).

### Gene expression analysis by quantitative real-time PCR

Quantitative real-time PCR (qRT-PCR) assays were performed using the Rotor-Gene^TM^ 6000 real-time PCR instrument (Corbett Research, Mortlake, Australia). All reactions were carried out in a 10 μl final volume containing 0.6 μl of forward and reverse primers, 1 μl of cDNA (about 10 wng of mRNA), and 5 μl Faststart Universal SYBR Green Master (ROX, Mannheim, Germany). Amplifications were performed according to the following conditions: 10 min initial polymerase activation at 95°C, then 40 cycles of denaturation at 95°C, 30 s, annealing at appropriate temperature (Table S1) for 30 s and extension at 72°C for 30 s. Relative quantification values for each target gene were calculated by the 2^–ΔΔCt^ method [49]. Gene expression levels were normalized using *18S* rRNA gene of *K. obovata* (acquired from NCBI, Gene Bank number AY289625.1) as the internal standard. The gene-specific primers and optimized reaction conditions used for qRT-PCR are listed in Table S1.

### Statistical analysis

Each experiment was repeated at least three times. All data were subjected to one-way analysis of variance (ANOVA) using the SPSS 13.0 (SPSS Chicago, IL, USA), and results were expressed as the mean ± SE. Difference at *P*<0.05 was considered significant between treatments.

### Ethics statement

State Administration of Jiulongjiang River Mangrove Nature Reserve in Fujian Province, China, gave permission to collect the mature propagules of *K. obovata* in this site for investigating the salt tolerance mechanism of the mangrove plants, and they confirmed that our studies did not involve endangered or protected species.

## Supporting Information

Table S1 (DOC)
