# Supplementary material for: Nitric Oxide Mediates Root K+/Na+ Balance in a Mangrove Plant, Kandelia obovata, by Enhancing the Expression of AKT1-Type K+ Channel and Na+/H+ Antiporter under High Salinity
Source: PLoS One. 2013 Aug 19;8(8):e71543. doi: 10.1371/journal.pone.0071543 (PMC3747234; doi:10.1371/journal.pone.0071543)
Supplement: Table S1 — (DOC) [file pone.0071543.s001.doc]

Table S1. Optimized primer sequences and reaction conditions used for gene cloning and real-time quantitative PCR of *SOS1*, *NHX1*, *HA1*, *VHA-c1*, *AKT1* and *18S rRNA* in *K. obovata*.

| Group and name | | Primer sequences | Purpose | Optimized conditions  (Tm / Amplicon length) |
| --- | --- | --- | --- | --- |
|  |  |  |  |  |
| *SOS1* | | | | |
|  | *SOS1* sense1 | 5'-GGCTBRGVTTYATWTTCAATGAYAC-3' | Gene cloning | 48.0^o^C/599 bp |
|  | *SOS1* antisense1 | 5'-AAMAAAYTGNGTRGTTGAWCCRTTM-3' |  |  |
|  | *SOS1* sense2 | 5'-ACACAGTGATAGAGATTGCCTTGAC-3' | qRT-PCR | 58.0^o^C/165 bp |
|  | *SOS1* antisense2 | 5'-GCAAACCTTGTTGACTTTCGCCCTT-3' |  |  |
| *NHX1* | | | | |
|  | *NHX1* sense1 | 5'-GTNTTYGGNGARGGWGTTGTNAATGAT-3' | Gene cloning | 52.4^o^C/445 bp |
|  | *NHX1* antisense1 | 5'-TGTCCADNGCATCCATNCCAACATANAG-3' |  |  |
|  | *NHX1* sense2 | 5'-TCAGTCATCTTAGTCTCAGTATCGC-3' | qRT-PCR | 58.0^o^C/148 bp |
|  | *NHX1* antisense2 | 5'-CGATCTGTTGAGTGCCTGCCAAAAT-3' |  |  |
| *HA1* | | | | |
|  | *HA1* sense1 | 5'-AAGGCWGCHCAYCTBGTNGAYAGCAC-3' | Gene cloning | 56.8^o^C/509 bp |
|  | *HA1* antisense1 | 5'-CCAGCHCKTGCCTCYTTNGGATCAG-3' |  |  |
|  | *HA1* sense2 | 5'-CTATTGGGAACTTCTGTATCTGCTC-3' | qRT-PCR | 56.0^o^C/159 bp |
|  | *HA1* antisense2 | 5'-CAGACAAGACTGTAGGCATGGCAAT-3' |  |  |
| *VHA-c1* | | | | |
|  | *VHA-c1* sense1 | 5'-AACBGCBCCVTTCTTCGGCTTBCT-3' | Gene cloning | 51.8^o^C/449 bp |
|  | *VHA-c1* antisense1 | 5'-CDCGRGAAGAVARRATRATDCCAAC-3' |  |  |
|  | *VHA-c1* sense2 | 5'-GGATTAACCCCAAAGCAAAGTCAT-3' | qRT-PCR | 55.0^o^C/179 bp |
|  | *VHA-c1* antisense2 | 5'-GAGAATAAGGATCATCCCAACAAAC-3' |  |  |
| *AKT1* | | | | |
|  | *AKT1* sense1 | 5'-ATGCTYCGWCTHTGGCGTCTBCGNA-3' | Gene cloning | 54.5^o^C/773 bp |
|  | *AKT1* antisense1 | 5'-CCAGTDACDAGDATGTAGAAGTCTG-3' |  |  |
|  | *AKT1* sense2 | 5'-AAGTTCAGGACAGATTCAGAGGGAC-3' | qRT-PCR | 56.0^o^C/134 bp |
|  | *AKT1* antisense2 | 5'-GACACCCCGTAAAACAAGTAAACCT-3' |  |  |
| *18S rRNA* | | | | |
|  | *18S rRNA* sense | 5'-AGCAAGCCTACGCTCTGGATACATT-3' | qRT-PCR | 56.0^o^C/167 bp |
|  | *18S rRNA* antisense | 5'-CGCAGTTGTTCGTCTTTCATAAATCCA-3' |  |  |
